# Persistent hyperparathyroidism after preemptive kidney transplantation

**DOI:** 10.1007/s10157-023-02371-9

**Published:** 2023-06-23

**Authors:** Manabu Okada, Tetsuhiko Sato, Yuki Hasegawa, Kenta Futamura, Takahisa Hiramitsu, Toshihiro Ichimori, Norihiko Goto, Shunji Narumi, Asami Takeda, Yoshihiko Watarai

**Affiliations:** 1 Department of Transplant Surgery and Transplant Nephrology, Japanese Red Cross Aichi Medical Center Nagoya Daini Hospital, 2-9 Myoken-cho, Showa-ku, Nagoya, Aichi 4668650 Japan; 2 Department of Diabetes and Endocrinology, Japanese Red Cross Aichi Medical Center Nagoya Daini Hospital, Showa-ku, Nagoya, Aichi Japan; 3 Department of Nephrology, Japanese Red Cross Aichi Medical Center Nagoya Daini Hospital, Showa-ku, Nagoya, Aichi Japan

**Keywords:** Hyperparathyroidism, Multivariate analysis, Preemptive kidney transplantation

## Abstract

**Background:**

Long-term dialysis vintage is a predictor of persistent hyperparathyroidism (HPT) after kidney transplantation (KTx). Recently, preemptive kidney transplantation (PKT) has increased. However, the incidence, predictors, and clinical implications of HPT after PKT are unclear. Here, we aimed to elucidate these considerations.

**Methods:**

In this retrospective cohort study, we enrolled patients who underwent PKT between 2000 and 2016. Those who lost their graft within 1 year posttransplant were excluded. HPT was defined as an intact parathyroid hormone (PTH) level exceeding 80 pg/mL or hypercalcemia unexplained by causes other than HPT. Patients were divided into two groups based on the presence of HPT 1 year after PKT. The primary outcome was the predictors of HPT after PKT, and the secondary outcome was graft survival.

**Results:**

Among the 340 consecutive patients who underwent PKT, 188 did not have HPT (HPT-free group) and 152 had HPT (HPT group). Multivariate logistic regression analysis revealed that pretransplant PTH level (*P* < 0.001; odds ratio [OR], 5.480; 95% confidence interval [CI], 2.070–14.50) and preoperative donor-estimated glomerular filtration rate (*P* = 0.033; OR, 0.978; 95% CI, 0.957–0.998) were independent predictors of HPT after PKT. Death-censored graft survival was significantly lower in the HPT group than that in the HPT-free group (90.4% vs. 96.4% at 10 years, *P* = 0.009).

**Conclusions:**

Pretransplant PTH levels and donor kidney function were independent predictors of HPT after PKT. In addition, HPT was associated with worse graft outcomes even after PKT.

**Supplementary Information:**

The online version contains supplementary material available at 10.1007/s10157-023-02371-9.

## Introduction

Hyperparathyroidism (HPT) is a typical complication of chronic kidney disease (CKD) often associated with mortality and various complications [1]. Although successful kidney transplantation (KTx) improves HPT to some extent [2], it often persists and adversely affects clinical outcomes even after KTx [3]. Long-term dialysis vintage is one of the main predictors of persistent HPT after KTx. Krinap et al. demonstrated that long-term dialysis vintage was significantly associated with persistent HPT in 640 patients with KTx [4]. Yamamoto et al. also reported dialysis vintage as a predictor of posttransplant HPT in a study of 520 patients [5]. Preemptive kidney transplantation (PKT), KTx performed before the initiation of chronic dialysis, has recently increased [6]. PKT is a superior treatment option in terms of both patient and graft outcomes [6]. However, the incidence, predictors, and clinical implications of HPT after PKT are unclear. Therefore, we conducted a retrospective cohort study to elucidate these considerations.

## Materials and methods

### Study design and subjects

Consecutive patients who underwent PKT between January 2000 and December 2016 at the Japanese Red Cross Aichi Medical Center, Nagoya Daini Hospital (Nagoya, Japan) were included. Data were collected on July 31, 2022. PKT was indicated for patients with Stage 5 CKD with an estimated glomerular filtration rate (eGFR) less than 15 mL/min/1.73 m^2^. The exclusion criteria were as follows: patients who underwent chronic dialysis before KTx, those who underwent parathyroidectomy before KTx, those for whom data were lacking, those who had lost their kidney graft within 1 year of KTx, and those who were under 16 years of age at the time of KTx. HPT was defined as intact parathyroid hormone (PTH) levels of > 80 pg/mL or hypercalcemia unexplained by causes other than HPT 1 year after KTx; intact PTH level has been reported to be ≤ 80 pg/mL in over 98% of healthy individuals irrespective of vitamin D status in both PTH assays used in this study [7]. Hypercalcemia was defined as total serum calcium (Ca) levels > 10.5 mg/dL.

Patients who met the inclusion criteria were divided into two groups based on the presence or absence of HPT: the HPT-free group, comprising patients without HPT, and the HPT group, comprising patients with HPT 1 year after PKT. Each patients’ sex, age, body mass index (BMI), diabetes mellitus status (%), hypertension status(%), original disease of CKD (%), parathyroid gland size (mm) (the parathyroid gland size of the recipients was routinely measured by ultrasound [US] before KTx), number of human leukocyte antigen (HLA) mismatches, positivity of donor-specific HLA antibody (DSA), treatment with vitamin D supplementation and calcimimetics before PKT, eGFR and laboratory data before and 1 year post-PKT, mean blood pressure (MBP) 1 year after PKT, and graft survival were documented. In addition, Ca deposition and interstitial fibrosis and tubular atrophy (IFTA) in the kidney graft were assessed by reviewing the reports of protocol biopsy performed 1 h and 1 year after KTx. IFTA was graded by an experienced transplant pathologist according to the Banff classification system. The primary outcome was the predictor of HPT after PKT, and the secondary outcome was death-censored graft survival. Pretransplant blood sample analyses were performed on all patients within 3 months before KTx, and posttransplant blood sample analyses were performed every month for 1 year after KTx and every second month thereafter. All samples were collected from fasting patients. The values of serum Ca and intact PTH obtained from blood samples 1 year after PKT were used for patient enrollment and classification. This study was conducted in accordance with the Strengthening the Reporting of Observational Studies in Epidemiology (STROBE) guidelines.

### Measurements

Serum Ca and phosphorus (P) levels were measured using standard methods. Intact PTH levels were measured using the following second-generation immunoassays: an electrochemical luminescence immunoassay (SRL, Tokyo, Japan, www.srl-group.co.jp, reference range [10–65 pg/mL]) or an enzyme immunoassay (TOSOH Company, Tokyo, Japan, www.tosoh.co.jp, reference range [9–80 pg/mL]). When serum albumin values were < 4.0 g/dL, all serum Ca values were corrected for serum albumin values as follows [8]:

$$ {\text{Corrected Ca }}\left( {{\text{mg}}/{\text{dL}}} \right)\, = \,{\text{measured total Ca }}\left( {{\text{mg}}/{\text{dL}}} \right)\, + \,0.{8} \times \left( {{4}.0\, - \,{\text{serum albumin }}\left[ {{\text{g}}/{\text{dL}}} \right]} \right). $$

The eGFR was evaluated using the creatinine equation provided by the Japanese Society of Nephrology [9].

### Immunosuppression

Immunosuppressive regimens included calcineurin inhibitors (cyclosporine or tacrolimus), mycophenolic acid, mizoribine, everolimus, and glucocorticoids. Basiliximab was used as induction therapy. In addition, rituximab administration or splenectomy was used as induction therapy in anti-donor antibody-positive patients after KTx, except in those with low antibody titers.

### Statistical analysis

Pearson’s Chi-square test was used to analyze nominal variables, and the Mann–Whitney* U* test was used for continuous variables. All results are presented as median (interquartile range [IQR]) because of their non-normal distribution, as confirmed by the Shapiro–Wilk normality test and histogram. The Spearman’s rank correlation coefficient was used to evaluate the correlations among the variables (Online Resource 1). Multivariate logistic regression analysis was performed to identify predictors of persistent HPT after PKT. Age [10], sex [11], diabetes mellitus [12], parathyroid gland size [13], serum Ca [14], P [15], and PTH levels [16] before PKT were included as covariates in the multivariate analysis; these factors have been associated with HPT in previous studies. In addition, preoperative donors’ eGFR, BMI, and hypertension were included in the multivariate analysis because of the heterogeneity between the HPT-free and HPT groups. Crude and multivariate-adjusted odds ratios (ORs) for HPT after PKT with intact PTH levels and preoperative donor eGFR categorized by tertiles were also examined. Kaplan–Meier survival curves and log-rank tests were used to estimate graft survival. Cox proportional hazards regression analysis was performed to evaluate the risk of death-censored graft loss. To assess the impact of HPT after PKT on graft survival, propensity score (PS) matching in a 1:1 ratio between the HPT-free and HPT groups was performed (Online Resources 2 and 3). A logistic regression model involving ten covariates was used to derive the PSs. These covariates included six continuous variables (recipient age, BMI, serum P, serum Ca, recipient eGFR, and MBP 1 year after KTx) and four nominal variables (recipient sex, ABO blood type incompatibility, diabetes mellitus, and preformed DSA). For sensitivity analysis, stratified hazard ratios (HRs) of HPT for graft loss were also analyzed using recipient age, BMI, serum Ca level, serum P level, MBP, ABO blood type, and sex (Online Resource 4). SPSS version 23.0 (IBM Corp., Armonk, NY, USA) and EZR version 1.40 [17] were used for the statistical analyses. Statistical significance was set at *P* < 0.05.

## Results

### Patient characteristics

**Fig. 1:**
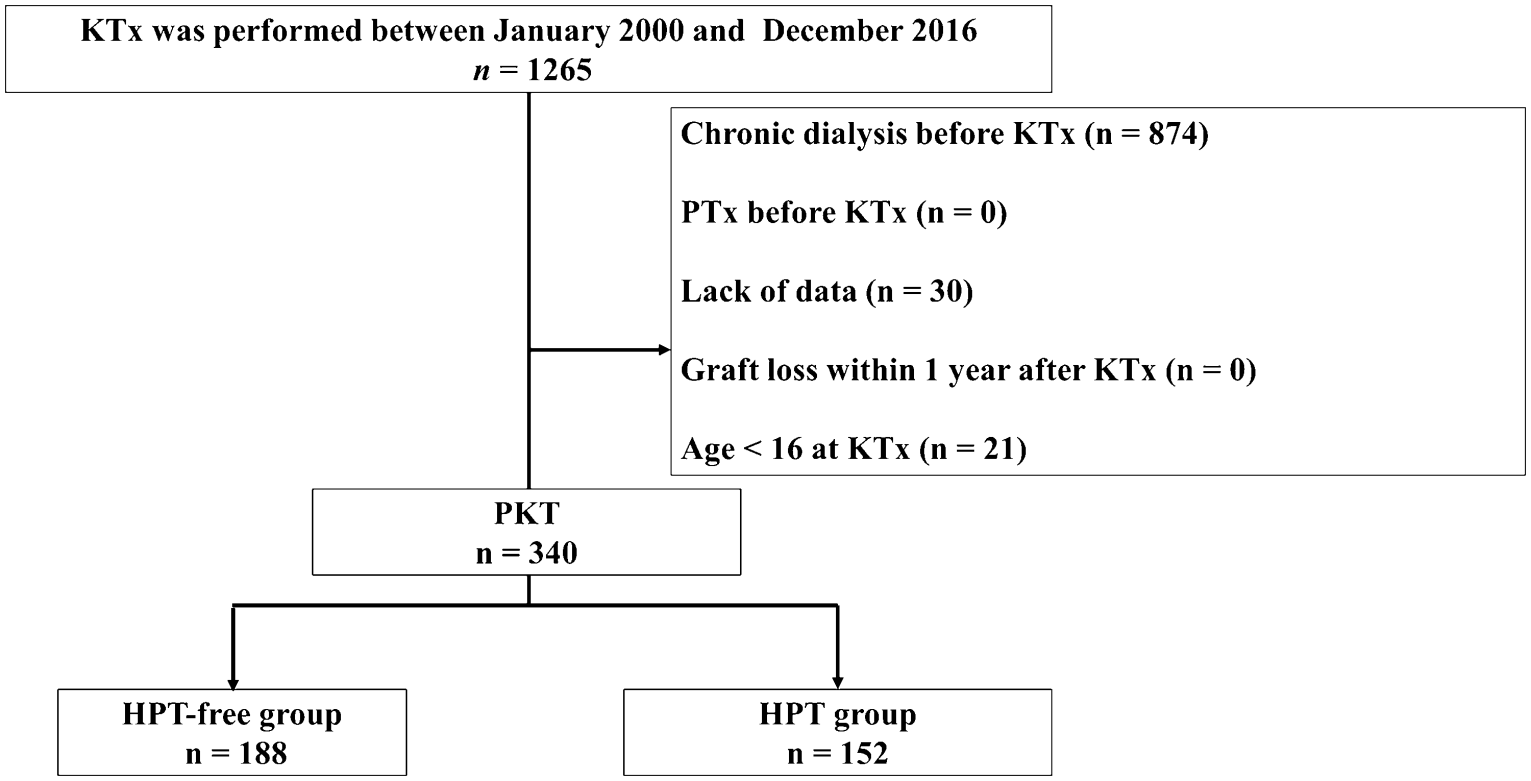
Study selection flowchart. *HPT* hyperparathyroidism, *PKT* preemptive kidney transplantation, *PTx* parathyroidectomy, *KTx* kidney transplantation

A total of 340 patients met the inclusion criteria (median observation period, 113 months [IQR, 86–146 months]). Of 340 patients, 188 and 152 were assigned to the HPT-free and HPT groups, respectively (Fig. 1). Patient characteristics are presented in Table 1. Significant differences were observed between both groups in donor age, donor hypertension, intact PTH level before PKT, and preoperative donor eGFR. In addition, serum Ca, intact PTH, and recipient eGFR 1 year after PKT were significantly different between the two groups. Other characteristics did not differ between the two groups (Table 1). Among the HPT group, two received parathyroidectomy and four received bisphosphonate treatment after PKT. None of the patients were administered calcimimetics during the follow-up period.

**Table 1:** Patient characteristics and clinical outcomes

	HPT free *N* = 188	HPT *N* = 152	*P* value
Baseline characteristics			
Recipient			
Age (years)	42 (31–55)	45 (36–56)	0.162
Sex (male, %)	118 (62.8)	86 (56.6)	0.247
Body mass index (kg/m^2^)	21.4 (19.6–24.2)	22.3 (19.6–24.8)	0.471
Diabetes mellitus (%)	38 (20.2)	25 (16.4)	0.374
Hypertension (%)	166 (91.2)	140 (93.3)	0.609
Parathyroid gland size (mm)	0.0 (0.0–3.8)	0.0 (0.0–5.0)	0.383
Vitamin D supplementation before PKT (%)	47 (25.0)	50 (32.9)	0.109
Original disease (%)			0.863
Glomerular disease/vasculitis	70 (37.2)	66 (43.4)	
Congenital/hereditary disease	35 (18.6)	24 (15.8)	
Diabetic nephropathy	25 (13.3)	16 (10.5)	
Nephrosclerosis	9 (4.8)	8 (5.3)	
Others	9 (4.8)	6 (3.9)	
Unknown	40 (21.3)	32 (21.1)	
Preoperative recipient eGFR (mL/min/1.73 m^2^)	8.0 (5.9–10.3)	7.8 (6.4–9.6)	0.895
Donor			
Age (years)	57 (50–63)	61 (53–67)	0.003*
Sex (male, %)	68 (36.2)	51 (33.6)	0.615
Body mass index (kg/m^2^)	22.7 (21.1–24.6)	22.3 (20.6–24.5)	0.178
Diabetes mellitus (%)	5 (2.7)	6 (3.9)	0.726
Hypertension (%)	33 (17.6)	44 (28.9)	0.018*
Preoperative donor eGFR (mL/min/1.73 m^2^)	73.4 (67.7–83.2)	71.0 (63.9–79.4)	0.003*
HLA AB mismatch	2 (2–3)	2 (1–3)	0.679
HLA DR mismatch	1 (1–2)	1 (1–2)	0.151
Preformed DSA (%)	11 (5.9)	6 (3.9)	0.423
ABO blood type incompatible KTx (%)	51 (27.1)	47 (30.9)	0.443
Laboratory data before PKT			
Corrected calcium (mg/dL)	9.0 (8.6–9.3)	8.9 (8.6–9.2)	0.099
Serum phosphorus (mg/dL)	4.9 (4.3–5.7)	4.9 (4.2–5.7)	0.716
Serum intact PTH (pg/mL)	293.5 (181.5–406.2)	378.0 (246.5–560.5)	< 0.001*
Lab data 1 year post-PKT			
Corrected calcium (mg/dL)	9.8 (9.6–10.0)	9.6 (9.4–9.8)	< 0.001*
Serum phosphorus (mg/dL)	3.5 (3.1–3.7)	3.3 (3.0–3.7)	0.230
Serum intact PTH (pg/mL)	55.5 (44.0–69.0)	100.0 (88.0–127.0)	< 0.001*
Recipient eGFR (mL/min/1.73m^2^)	47.3 (41.4–54.6)	42.1 (35.7–48.6)	< 0.001*
Urine calcium (mg/dL)	2.6 (1.5–4.7)	2.6 (1.5–4.6)	0.732
Urine phosphorus (mg/dL)	25.5 (19.0–34.0)	26.0 (20.0–37.3)	0.159
MBP 1 year post-PKT (mmHg)	92.7 (83.3–99.9)	95.3 (87.3–102.6)	0.087
Clinical outcomes			
Death (%)	4 (2.1)	6 (3.9)	NA
Graft loss (%)	11 (5.9)	17 (11.2)	NA
Follow-up period (months)	117 (89–161)	107 (83–134)	0.012*

Data for continuous variables are presented as median (interquartile range)

*DSA* donor-specific HLA antibody, *eGFR* estimated glomerular filtration rate, *HPT* hyperparathyroidism, *KTx* kidney transplantation, *MBP* mean blood pressure, *NA* not applicable, *PTH* parathyroid hormone, *PKT* preemptive kidney transplantation

*P value < 0.05

The parathyroid gland size was defined as 0 when the parathyroid gland was not detected by echography

### Predictors of HPT after PKT

**Fig. 2:**
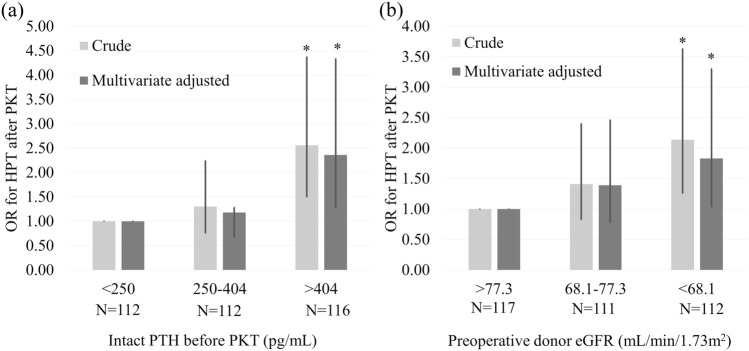
ORs and 95% CIs for HPT after PKT according to the categories of pretransplant intact PTH levels and preoperative donor eGFR using crude and multivariable-adjusted logistic models. **a** ORs according to the categories of pretransplant intact PTH levels. The multivariable-adjusted analysis included recipient age, recipient sex, diabetes mellitus, pretransplant serum Ca and *P* levels, parathyroid gland size, donor age, donor sex, and preoperative donor eGFR. **b** ORs according to categories of preoperative donor eGFR. The multivariable-adjusted analysis included recipient age, recipient sex, diabetes mellitus, pretransplant serum intact PTH, Ca and P levels, parathyroid gland size, donor age, and donor sex. *P < 0.05. *Ca* calcium, *95% CI* 95% confidence interval, *eGFR* estimated glomerular filtration rate, *HPT* hyperparathyroidism, *OR* odds ratio, *PKT* preemptive kidney transplantation, *PTH* parathyroid hormone

The univariate logistic regression analysis demonstrated that log-intact PTH levels before PKT (*P* < 0.001; OR, 5.450; 95% confidence interval [CI] 2.380–12.50), donor age (*P* = 0.006; OR, 1.030; 95% CI 1.010–1.060), donor hypertension (*P* = 0.013; OR, 1.910; 95% CI 1.140–3.200), and preoperative donor eGFR (*P* = 0.002, OR, 0.971; 95% CI 0.953–0.989) were significantly associated with HPT after PKT (Table 2). Multivariate logistic regression analysis revealed that log-intact PTH before PKT (*P* < 0.001; OR, 5.500; 95% CI 2.090–14.50) and preoperative donor eGFR (*P* = 0.034; OR, 0.977; 95% CI 0.957–0.998) were significantly associated with HPT after PKT (Table 2). Figure 2 shows the crude and multivariate-adjusted ORs for HPT after PKT associated with the categories of intact PTH before PKT and the preoperative donor eGFR values. The multivariate-adjusted OR for HPT after PKT significantly increased when intact PTH levels exceeded 404.0 pg/mL (OR, 2.360; 95% CI 1.280–4.330) (Fig. 2a). Conversely, a significant increase in the multivariate adjusted OR of HPT after PKT was observed when preoperative donor eGFR was lower than 68.1 mL/min/1.73 m^2^ (OR, 1.830; 95% CI 1.010–3.300) (Fig. 2b).

**Table 2:** Logistic regression for HPT after PKT

Factors	Univariate			Multivariate		
	OR	95% CI	*P* value	OR	95% CI	*P* value
Recipient age (yeas)	1.010	0.995–1.030	0.192	1.010	0.994–1.030	0.200
Male recipient	0.773	0.500–1.200	0.247	0.861	0.507–1.460	0.579
Recipient diabetes mellitus	0.777	0.445–1.360	0.375	0.734	0.391–1.460	0.336
Serum Ca before PKT (mg/dL)	0.818	0.601–1.110	0.203	1.100	0.756–1.610	0.614
Serum P before PKT (mg/dL)	0.966	0.810–1.150	0.700	0.941	0.765–1.160	0.568
Log intact PTH before PKT (pg/mL)	5.450	2.380–12.50	< 0.001*	5.500	2.090–14.50	< 0.001*
Parathyroid gland size (mm)	1.040	0.984–1.100	0.164	1.020	0.958–1.080	0.549
Donor age (years)	1.030	1.010–1.060	0.006*	1.000	0.976–1.030	0.800
Male donor	0.891	0.569–1.400	0.615	0.770	0.449–1.320	0.342
Donor hypertension	1.910	1.140–3.200	0.013*	1.740	0.960–3.160	0.068
Donor body mass index (kg/m^2^)	0.959	0.890–1.030	0.272	0.938	0.862–1.020	0.136
Preoperative donor eGFR (mL/min/1.73 m^2^)	0.971	0.953–0.989	0.002*	0.977	0.957–0.998	0.034*

*Ca* calcium, *95% CI* 95% confidence interval, eGFR estimated glomerular filtration rate, *HPT* hyperparathyroidism, *PTH* intact parathyroid hormone, *Log* logarithm, *OR* odds ratio, *P* phosphorus, *PKT* preemptive kidney transplantation

^*^P value < *0.05*

### Graft survival

**Fig. 3:**
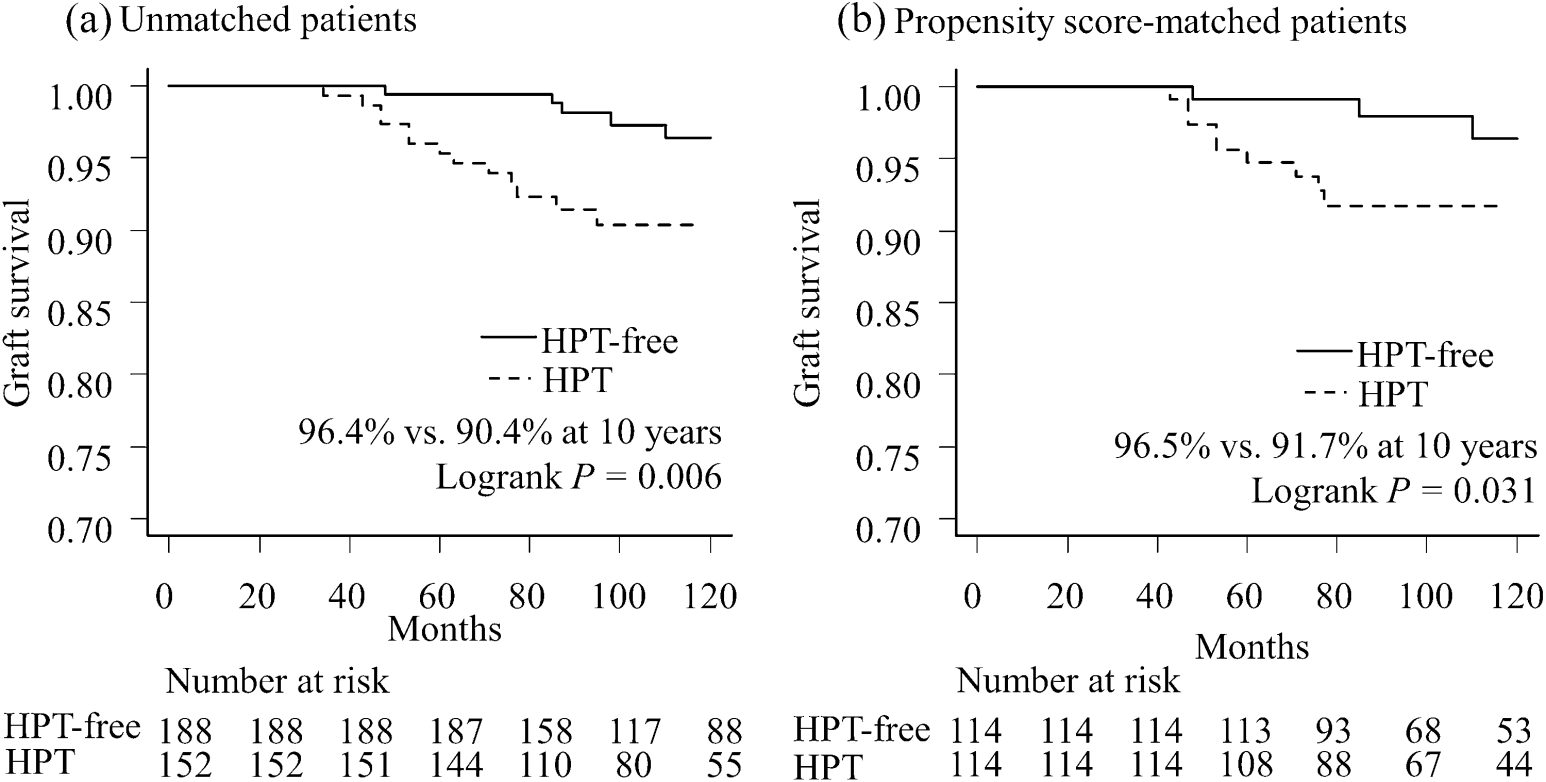
Death-censored graft survival curves according to status of hyperparathyroidism. *HPT* hyperparathyroidism

Graft loss was observed in 28 patients (6.3% vs. 10.7% in the HPT-free and HPT groups, respectively) (Table 1). Death-censored graft survival in the HPT group was significantly lower than that in the HPT-free group (90.4% vs. 96.4% at 10 years, *P* = 0.006) (Fig. 3a). Even after PS matching of the 114 recipients from each group, the death-censored graft survival of HPT recipients was inferior to that of HPT-free recipients (91.7% vs. 96.5% at 10 years, *P* = 0.031) (Fig. 3b). Univariate Cox proportional hazards analysis revealed that HPT after PKT was significantly associated with death-censored graft loss (*P* = 0.009; HR, 2.840; 95% CI 1.304–6.182) (Table 3). In addition, the Cox proportional hazards model adjusted by PS matching revealed significantly higher risk of death-censored graft loss in the HPT group than that in the HPT-free group (*P* = 0.039; HR, 3.047; 95% CI 1.057–8.787) (Table 3). Even in the stratified analysis, although it was not necessarily statistically significant in all strata due to a decrease in the number of events in each group, HPT tended to increase the risk of graft loss overall (Online Resource 4).

**Table 3:** Cox regression analysis for death-censored graft loss of the HPT group with reference to the HPT-free group

Method	HR	95% CI	*P* value
Unadjusted (*N* = 340)	2.840	1.304–6.182	0.009*
Propensity score matching (*N* = 228)	3.047	1.057–8.787	0.039*

CI confidence interval, *HPT* hyperparathyroidism, *HR* hazard ratio

*P value < 0.05

### Graft biopsy findings

Protocol graft biopsies were performed in 335 and 325 patients 1 h, and 1 year after PKT, respectively. There was no significant difference in Ca deposition or IFTA between the two groups at 1 h post-PKT. However, both the incidence of Ca deposition and the severity of IFTA were significantly higher in the HPT group than those in the HPT-free group in kidney grafts 1 year post-PKT (Table 4).

**Table 4:** Calcium deposition and IFTA in kidney graft

	HPT free	HPT	*P* value
Protocol biopsy one hour post-PKT	*N* = 183	*N* = 152	
Calcium deposition (%)	0 (0.0)	0 (0.0)	1.000
IFTA (%)			0.090
I	4 (2.2)	10 (6.6)	
II	0 (0.0)	1 (0.7)	
III	0 (0.0)	0 (0.0)	
Protocol biopsy one year post-PKT	*N* = 176	*N* = 149	
Calcium deposition (%)	7 (4.0)	14 (9.4)	0.048*
IFTA (%)			0.045*
I	40 (22.7)	38 (25.5)	
II	8 (4.5)	16 (10.7)	
III	0 (0.0)	2 (1.3)	

*HPT* hyperparathyroidism, *IFTA* interstitial fibrosis and tubular atrophy, *PKT* preemptive kidney transplantation

*P value < 0.05

## Discussion

We hypothesized that the incidence of persistent HPT after PKT would be considerably lower because there is no dialysis vintage. However, high PTH levels were observed in over 40% of the patients even 1 year after PKT in the current study. Multivariate logistic regression analysis demonstrated that pretransplant PTH level and preoperative donor eGFR were predictors of HPT after PKT. In contrast, parathyroid gland size was not a predictor of HPT after PKT in the present study, which was inconsistent with previous reports [13]. This discrepancy is likely due to the limited accuracy of US imaging of the parathyroid glands [18].

The association between high pretransplant PTH levels and HPT after PKT demonstrated in the current study is consistent with previous reports in patients who underwent KTx, not limited to PKT. Kirnap et al. reported that intact PTH levels are a risk factor for persistent HPT after KTx [4]. Another study by Yamamoto et al. showed that intact pretransplant PTH levels correlated with HPT after KTx [5]. In addition, Sutton et al. demonstrated that intact PTH levels of > 300 pg/mL before KTx were associated with the development of HPT after KTx [16]. These findings highlight the importance of managing CKD mineral and bone disorders before KTx to prevent persistent HPT.

To the best of our knowledge, the significant association observed between a lower preoperative donor eGFR and persistent HPT after KTx is a novel finding. Kidney graft function depends, to some extent, on donor GFR [19, 20]. In addition, vitamin D status is associated with GFR [21] and plays a central role in HPT [22]. Taken together, it is reasonable to conclude that a low donor GFR contributes to the progression of posttransplant HPT through low kidney graft function and vitamin D deficiency. In other words, sufficient donor kidney function is important to prevent HPT after KTx.

In previous reports, posttransplant HPT was associated with poor kidney graft outcomes [3, 23, 24]. In this study, which focused only on patients who underwent PKT, graft survival was lower in patients with HPT than that in patients who were HPT-free. Furthermore, the inferiority in graft survival of the HPT group did not change even after PS matching for several known risk factors. This result indicates the importance of posttransplant monitoring of PTH and the recognition of persistent HPT as a risk factor for graft loss, regardless of dialysis vintage. Therefore, the necessity of posttransplant HPT treatment should be discussed even after PKT. Although the mechanisms by which HPT worsens graft survival are unclear, excess PTH has been associated with pathological fibrosis [25] and renal/vascular calcification [26–28]. In the current study, both the incidence of Ca deposition and the severity of IFTA on kidney grafts were higher in the HPT group than those in the HPT-free group, which may have affected long-term graft survival. Compared to the HPT-free group, the serum Ca levels were lower and the urine Ca levels were similar in the HPT group. In addition, most of the calcifications in the kidney grafts occurred in the epithelial cells and interstitium and not in the tubular lumen. Therefore, those lesions may be due to factors other than Ca concentration. Recently, various factors such as inflammatory cytokines, endoplasmic reticulum stress, autophagy dysfunction, mitochondrial dysfunction, and abnormal molecular signaling pathways have been reported to be involved in soft tissue calcification in addition to calcium and phosphorus concentrations. The mechanism of calcification of kidney grafts is unclear; however, it may be the result of the complex interaction of these multifactorial variables [29, 30].

This study has several limitations. First is its retrospective design. Second, it is a single-center study. Third is the inherent possibility of unmeasured confounders. Fourth, there may be selection bias. Fifth is the absence of data regarding endogenic vitamin D status, fibroblast growth factor 23, and other bone biomarkers. Further studies with larger sample sizes are required to validate our findings.

## Conclusions

The incidence of persistent HPT 1 year after PKT is 40%, and pretransplant PTH levels and donor eGFR are the predictors. Monitoring and management of PTH levels even after PKT are equally important because HPT is associated with poor graft survival.

## Supplementary Information

Below is the link to the electronic supplementary material.

Supplementary file1 Correlation analysis using Spearman’s rank correlation coefficient (PDF 86 KB)

Supplementary file2 (TIF 1317 KB)

Supplementary file3 (PDF 117 KB)

Supplementary file4 (TIF 913 KB)

Supplementary file5 (PDF 123 KB)

## Data Availability

The datasets generated and/or analyzed in the current study are available from the corresponding author upon reasonable request.
