# Supplementary material for: Persistent hyperparathyroidism after preemptive kidney transplantation
Source: Clin Exp Nephrol. 2023 Jun 23;27(10):882–9. doi: 10.1007/s10157-023-02371-9 (PMC10504143; doi:10.1007/s10157-023-02371-9)
Supplement: Supplementary file 1 — Supplementary file1 Correlation analysis using Spearman’s rank correlation coefficient (PDF 86 KB) [file 10157_2023_2371_MOESM1_ESM.pdf]

Persistent hyperparathyroidism after preemptive kidney transplantation
Clinical and Experimental Nephrology
Manabu Okada, Tetsuhiko Sato, et al.

Corresponding author: Manabu Okada, M.D., Ph.D.
Department of Transplantation and Endocrine Surgery,
Japanese Red CrossAichi Medical Center Nagoya Daini Hospital, 2-9 Myoken-cho,
Showa-ku, Nagoya, Japan 4668650
Tel: 81-528321121 Fax: 81-528321130 E-mail: ubanam@nagoya2.jrc.or.jp

Spearman's rank correlation coefficient

		Recipient age (years)	Recipient sex	Recipient diabetes mellitus	Donor sex	Donor Age (years)	Preoperative donor GFR (mL/min/1.73m2)	Parathyroid gland size (mm)	Serum Ca before PKT (mg/dL)	Serum P before PKT (mg/dL)	Serum intact PTH before PKT (pg/mL)	Donor BMI (kg/m^2)	Donor hypertension
**Recipient age (years)**	**Correlation coefficient**	1.000	0.016	.303**	-0.039	.235**	-0.079	0.084	-.139*	-.147**	-0.086	-0.011	0.027
	***P* value**		0.768	0.000	0.471	0.000	0.145	0.124	0.010	0.007	0.113	0.838	0.613
	**Number**	340	340	340	340	340	340	340	340	340	340	337	340
**Recipient sex**	**Correlation coefficient**	0.016	1.000	.127*	-.408**	-0.030	0.023	-0.045	-0.034	-.128*	-.260**	-.135*	-0.075
	***P* value**	0.768		0.019	0.000	0.587	0.666	0.408	0.531	0.018	0.000	0.013	0.170
	**Number**	340	340	340	340	340	340	340	340	340	340	337	340
**Recipient diabetes mellitus**	**Correlation coefficient**	.303**	.127*	1.000	-.128*	0.021	-0.006	-0.036	0.007	0.014	-.114*	-0.016	0.031
	***P* value**	0.000	0.019		0.018	0.706	0.914	0.510	0.901	0.799	0.036	0.765	0.565
	**Number**	340	340	340	340	340	340	340	340	340	340	337	340
**Donor sex**	**Correlation coefficient**	-0.039	-.408**	-.128*	1.000	-0.012	-0.008	0.104	0.063	0.053	.130*	.241**	0.030
	***P* value**	0.471	0.000	0.018		0.832	0.878	0.055	0.247	0.327	0.016	0.000	0.579
	**Number**	340	340	340	340	340	340	340	340	340	340	337	340
**Donor Age (years)**	**Correlation coefficient**	.235**	-0.030	0.021	-0.012	1.000	-.395**	-0.048	-.141**	-0.050	.131*	-0.009	.375**
	***P* value**	0.000	0.587	0.706	0.832		0.000	0.377	0.009	0.356	0.016	0.874	0.000
	**Number**	340	340	340	340	340	340	340	340	340	340	337	340
**Preoperative donor GFR (mL/min/1.73m2)**	**Correlation coefficient**	-0.079	0.023	-0.006	-0.008	-.395**	1.000	-0.004	0.067	0.019	-0.096	-0.091	-.195**
	***P* value**	0.145	0.666	0.914	0.878	0.000		0.945	0.218	0.733	0.076	0.096	0.000
	**Number**	340	340	340	340	340	340	340	340	340	340	337	340
**Parathyroid gland size (mm)**	**Correlation coefficient**	0.084	-0.045	-0.036	0.104	-0.048	-0.004	1.000	-0.101	0.090	0.062	0.093	-0.055
	***P* value**	0.124	0.408	0.510	0.055	0.377	0.945		0.062	0.096	0.251	0.088	0.310
	**Number**	340	340	340	340	340	340	340	340	340	340	337	340
**Serum Ca before PKT (mg/dL)**	**Correlation coefficient**	-.139*	-0.034	0.007	0.063	-.141**	0.067	-0.101	1.000	-.161**	-.400**	-0.024	-0.083
	***P* value**	0.010	0.531	0.901	0.247	0.009	0.218	0.062		0.003	0.000	0.663	0.125
	**Number**	340	340	340	340	340	340	340	340	340	340	337	340
**Serum P before PKT (mg/dL)**	**Correlation coefficient**	-.147**	-.128*	0.014	0.053	-0.050	0.019	0.090	-.161**	1.000	.135*	-0.022	0.002
	***P* value**	0.007	0.018	0.799	0.327	0.356	0.733	0.096	0.003		0.013	0.689	0.978
	**Number**	340	340	340	340	340	340	340	340	340	340	337	340
**Serum intact PTH before PKT (pg/mL)**	**Correlation coefficient**	-0.086	-.260**	-.114*	.130*	.131*	-0.096	0.062	-.400**	.135*	1.000	0.038	0.078
	***P* value**	0.113	0.000	0.036	0.016	0.016	0.076	0.251	0.000	0.013		0.492	0.153
	**Number**	340	340	340	340	340	340	340	340	340	340	337	340
**Donor BMI (kg/m^2)**	**Correlation coefficient**	-0.011	-.135*	-0.016	.241**	-0.009	-0.091	0.093	-0.024	-0.022	0.038	1.000	.150**
	***P* value**	0.838	0.013	0.765	0.000	0.874	0.096	0.088	0.663	0.689	0.492		0.006
	**Number**	337	337	337	337	337	337	337	337	337	337	337	337
**Donor hypertension**	**Correlation coefficient**	0.027	-0.075	0.031	0.030	.375**	-.195**	-0.055	-0.083	0.002	0.078	.150**	1.000
	***P* value**	0.613	0.170	0.565	0.579	0.000	0.000	0.310	0.125	0.978	0.153	0.006	
	**Number**	340	340	340	340	340	340	340	340	340	340	337	340
