# Supplementary figures and images for: Persistent hyperparathyroidism after preemptive kidney transplantation
Source: Clin Exp Nephrol. 2023 Jun 23;27(10):882–9. doi: 10.1007/s10157-023-02371-9 (PMC10504143; doi:10.1007/s10157-023-02371-9)

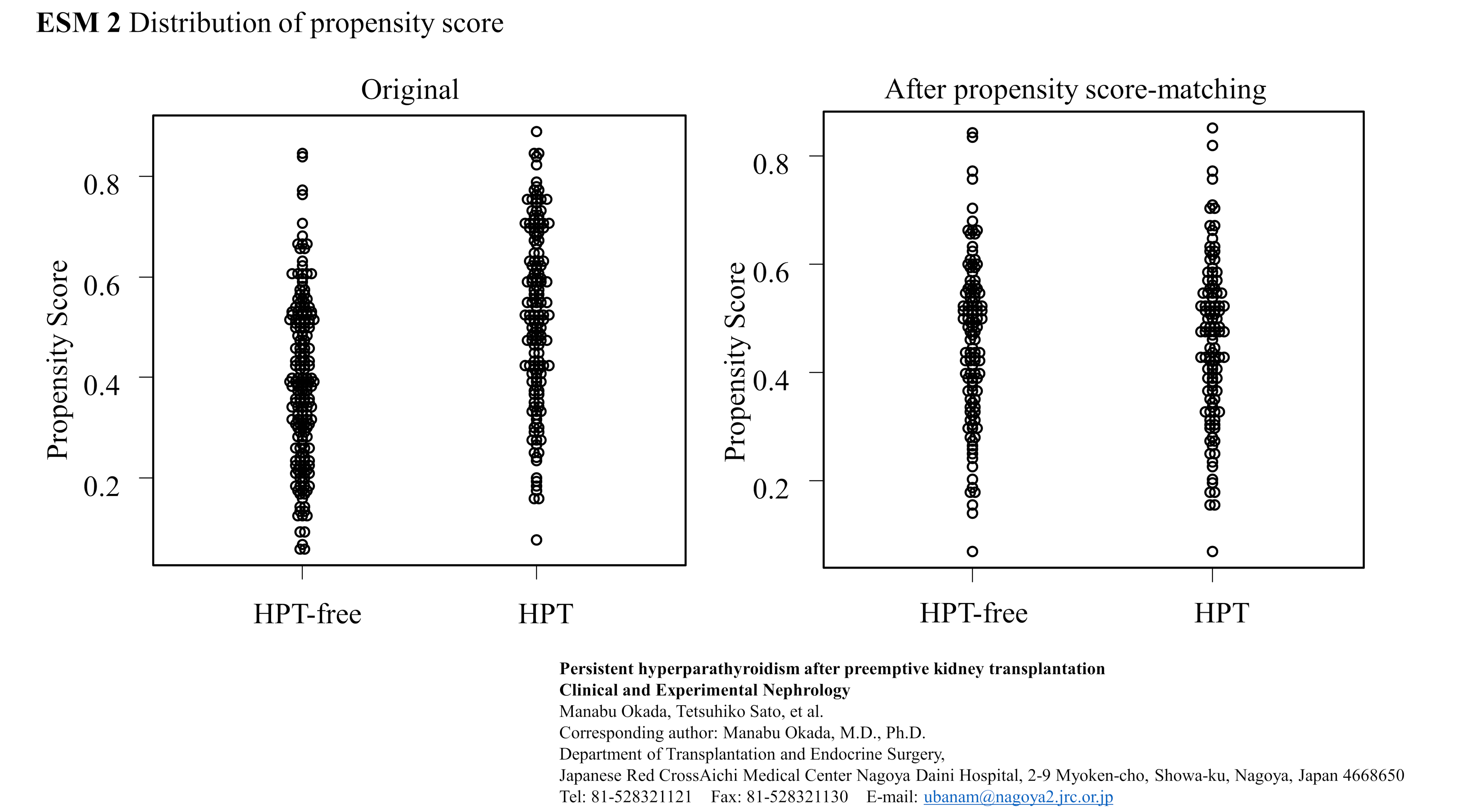

Supplement: Supplementary file 2 — Supplementary file2 (TIF 1317 KB) [file 10157_2023_2371_MOESM2_ESM.tif]

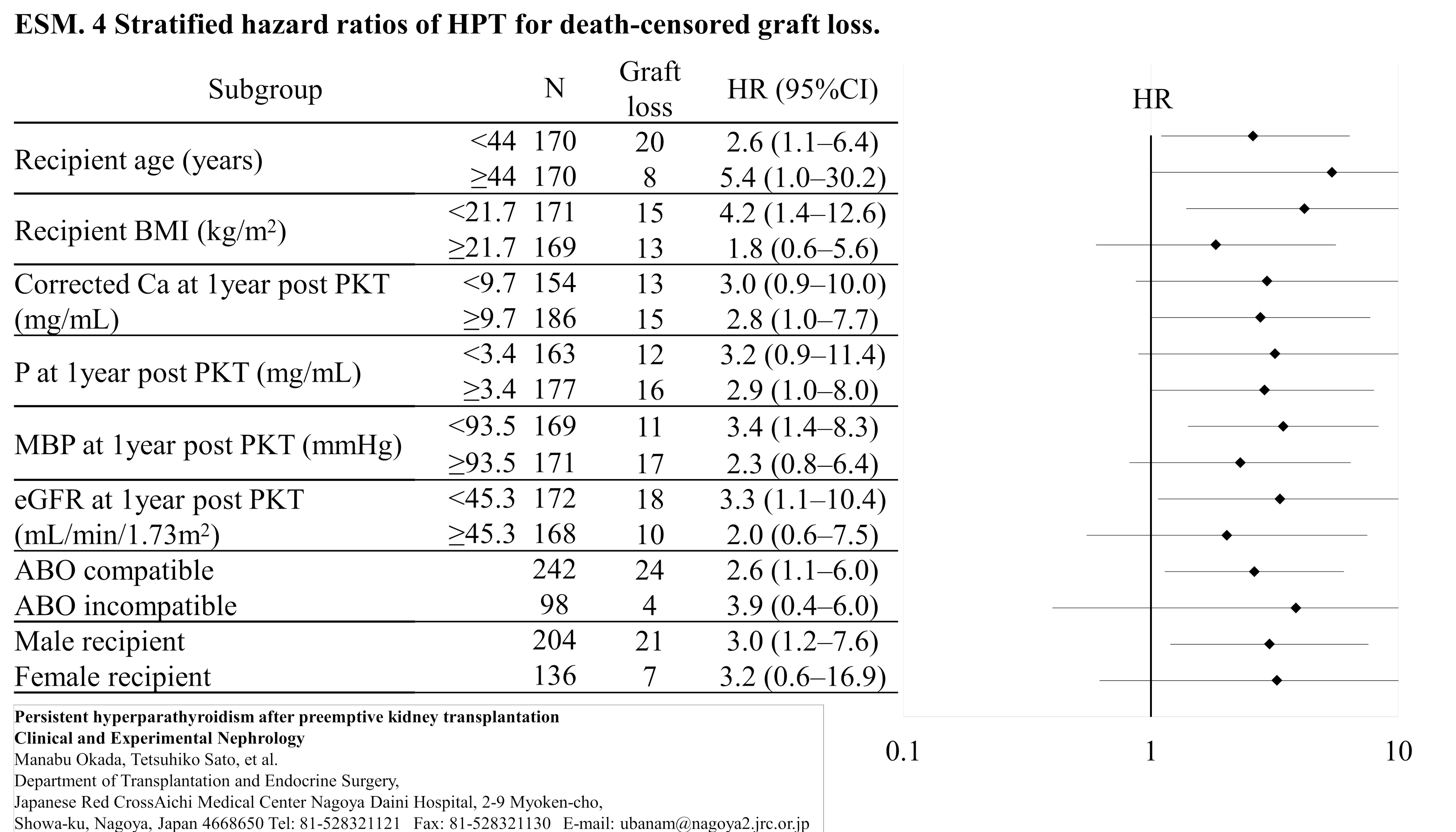

Supplement: Supplementary file 4 — Supplementary file4 (TIF 913 KB) [file 10157_2023_2371_MOESM4_ESM.tif]
