# Supplementary material for: Persistent hyperparathyroidism after preemptive kidney transplantation
Source: Clin Exp Nephrol. 2023 Jun 23;27(10):882–9. doi: 10.1007/s10157-023-02371-9 (PMC10504143; doi:10.1007/s10157-023-02371-9)
Supplement: Supplementary file 3 — Supplementary file3 (PDF 117 KB) [file 10157_2023_2371_MOESM3_ESM.pdf]

Persistent hyperparathyroidism after preemptive kidney transplantation

Clinical and Experimental Nephrology

Manabu Okada, Tetsuhiko Sato, et al.

Corresponding author: Manabu Okada, M.D., Ph.D.

Department of Transplantation and Endocrine Surgery,

Japanese Red Cross Aichi Medical Center Nagoya Daini Hospital, 2-9 Myoken-cho,

Showa-ku, Nagoya, Japan 4668650

Tel: 81-528321121 Fax: 81-528321130 E-mail: ubanamadako@yahoo.co.jp

ESM 3. Patient characteristics after propensity score matching

	HPT-free N = 114	HPT N = 114	*P* -value	SMD
Recipient age (years)	47 (35–57)	44 (35–59)	0.831	0.037
Male recipient (%)	79 (69.3)	70 (61.4)	0.266	0.166
Body mass index (kg/m^2)	21.6 (19.8–24.4)	21.7 (19.4–24.9)	0.689	0.036
Diabetes mellitus (%)	20 (17.5)	22 (19.3)	0.864	0.045
Preformed DSA (%)	6 (5.3)	4 (3.5)	0.746	0.086
ABO blood type incompatible KTx (%)	37 (32.5)	33 (28.9)	0.667	0.076
Lab data one year post PKT				
Serum calcium (mg/dL)	9.8 (9.6–9.9)	9.7 (9.4–9.9)	0.228	0.044
Serum phosphorus (mg/dL)	3.3 (3.0–3.6)	3.4 (3.0–3.8)	0.417	0.103
Intact PTH (pg/mL)	51.5 (43.3–71.0)	96.5 (86.3–118.0)	<0.001*	1.403
Recipient eGFR (mL/min/1.73m^2)	44.4 (38.7–48.8)	44.7 (38.4–51.1)	0.660	0.032
MBP one year post PKT (mmHg)	95.0 (85.0–100.7)	94.0 (83.8–101.9)	0.583	0.112

Data for continuous variables are presented as median (interquartile range).

DSA , donor-specific HLA antibody; *eGFR* , estimated glomerular filtration rate; *HPT* , hyperparathyroidism; *MBP* , mean blood pressure; *PKT* , preemptive kidney transplantation; *PTH* , parathyroid hormone; *KTx* , kidney transplantation; *SMD* , standardized mean difference.

**P* -value < 0.05
